# A Case of Dysembryoplastic Neuroepithelial Tumor in an Adolescent Male

**DOI:** 10.7759/cureus.13917

**Published:** 2021-03-16

**Authors:** Marcel Yibirin, Diana De Oliveira, Isabella Suarez, Gabriela Lombardo, Carlos Perez

**Affiliations:** 1 Department of Infectious Diseases, Infection Control, and Employee Health, University of Texas Monroe Dunaway (MD) Anderson Cancer Center, Houston, USA; 2 Department of Research, Foundation for Clinic, Public Health, and Epidemiological Research of Venezuela (FISPEVEN), Caracas, VEN; 3 Department of Research, Luis Razetti School of Medicine, Central University of Venezuela, Caracas, VEN; 4 Division of Multiple Sclerosis and Neuroimmunology, Department of Neurology, University of Texas Health Science Center at Houston, Houston, USA

**Keywords:** brain, drug resistance, epilepsy, glial fibrillary acidic protein, neoplasms, neuroepithelial

## Abstract

Dysembryoplastic neuroepithelial tumors (DNETs) are benign mixed glioneuronal neoplasms that frequently occur in children and young adults. We present the case of a 17-year-old male who arrived at the hospital following seizure-like activity. A magnetic resonance imaging (MRI) scan showed a 10 x 8 x 10 mm, oval-shaped, non-enhancing, well-defined mass within the right hippocampus. The patient underwent a transcortical approach via the middle temporal gyrus for resection of the mass; histopathological examination demonstrated the presence of round, uniform cells in an extensively myxoid background with diffuse reactivity to glial fibrillary acidic protein (GFAP). DNETs are considered benign, non-recurring lesions. Complete surgical resection is associated with a seizure-free outcome in 80% to 100% of cases.

## Introduction

Dysembryoplastic neuroepithelial tumors (DNETs) are benign mixed glioneuronal neoplasms that frequently occur in children and young adults [1], especially in the age group of 10-14 years, which is described to present the most significant peak; the incidence is higher in males than females [2]. They were described for the first time in 1998 by Damas-Duport [3] and are characterized histologically by the presence of oligodendrocyte-like cells [4]. DNETs may present with chronic drug resistance seizures, the most commonly are partial complex seizures, associated with headache and papilledema in some cases [5]. We report the case of a patient, without a previous history of seizures, who arrived at the hospital after experiencing two episodes of generalized tonic-clonic seizures. After performing imaging studies, which showed a well-defined mass, the tumor was resected and the histopathological examination suggested a DNET.

## Case presentation

A 17-year-old adopted male with a history of bipolar and generalized anxiety disorders, on treatment with oxcarbazepine, presented to the hospital following a witnessed episode of generalized tonic-clonic seizures. He was reportedly confused for 20-30 minutes after regaining consciousness. He sustained right-sided head trauma with associated subconjunctival hemorrhage secondary to the fall and was transferred to an outside hospital where he had a second witnessed seizure. The patient was then transferred to our hospital for a higher level of care.

Upon admission, he was oriented to time, place, and person. His blood pressure was 128/72 mmHg and oxygen saturation was 88% on room air. The rest of his vital signs were within normal limits. On neurologic examination, his speech was dysarthric, he had short-term memory loss, and a brisk right patellar reflex. The remainder of the initial examination was normal.

Routine laboratory testing, including a complete blood count and a metabolic panel were normal. Ethanol, salicylate, and acetaminophen levels were negative. A serum oxcarbazepine level was 38 mcg/mL (reference range: negative).

**Figure 1:**
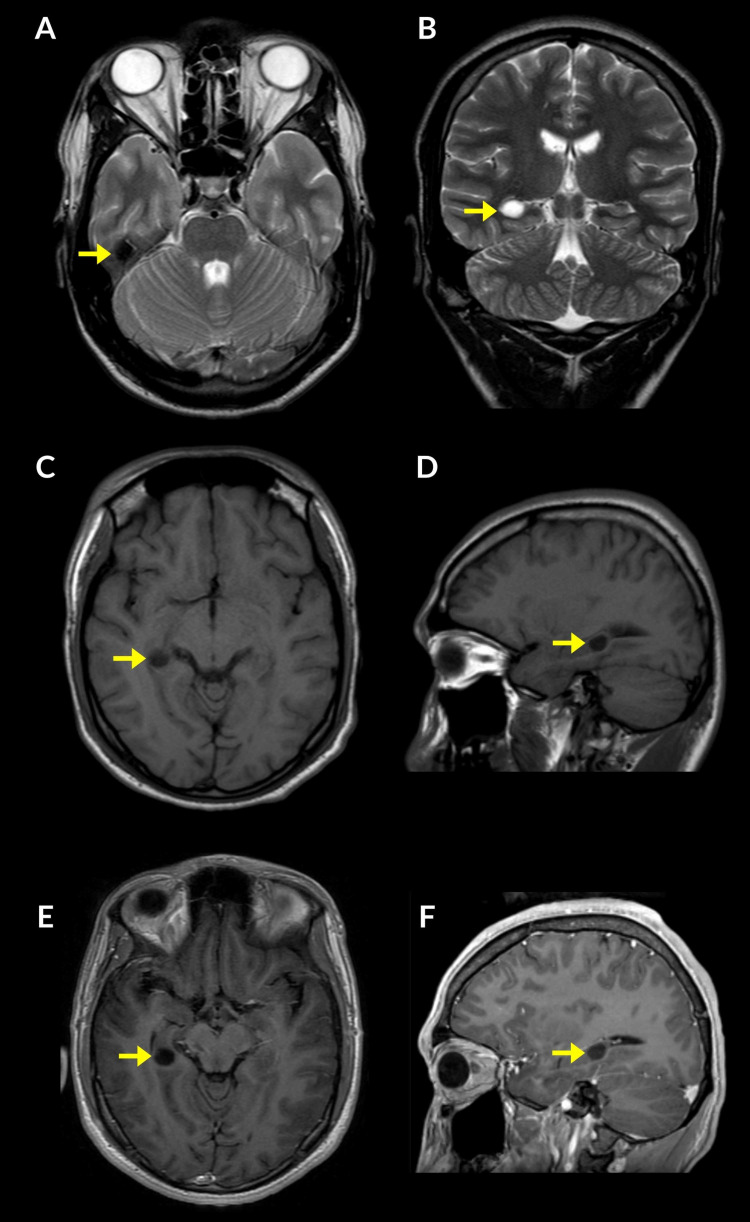
Brain magnetic resonance imaging (MRI) scan in a patient with DNET. Axial (A) and coronal (B) T2-weighted; pre-contrast axial (C) and sagittal (D) T1-weighted; and post-contrast axial (E) and sagittal (F) T1-weighted images showing the presence of an oval-shaped well-defined lesion in the body of the right hippocampus, which measures 10 x 8 x 10 mm in diameter (yellow arrows).

A brain magnetic resonance imaging (MRI) scan was obtained, which showed a 10 x 8 x 10 mm, oval-shaped, non-enhancing, well-defined mass within the body of the right hippocampus, causing superior displacement of the temporal horn of the lateral ventricle. A smaller second cystic lesion of 5 mm with similar characteristics was noted in the medial aspect of the inferior left temporal gyrus (Figure 1).

**Figure 2:**
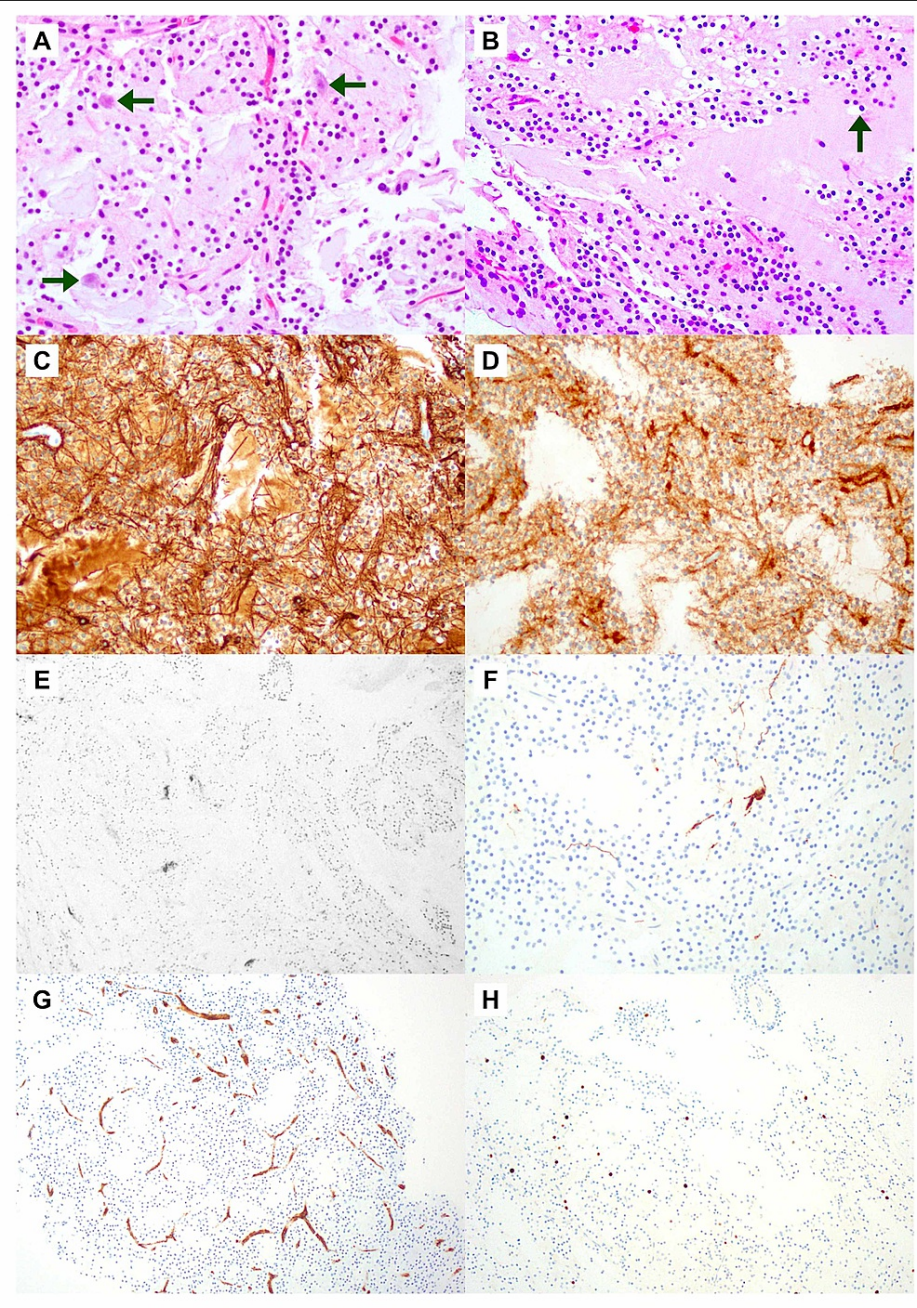
Immunohistochemistry results of surgical biopsy in a patient with DNET. (A, B) Hematoxylin-eosin staining (x100) showing uniform, round oligodendroglioma-like cells in an extensively myxoid background (green arrows), with delicate vasculature and scattered floating neurons. Various stains for (C) glial fibrillary acid protein (GFAP), (D) synaptophysin, (E) chromogranin, marker for neuroendocrine tumor - negative in this case, (F) phosphorylated neurofilament (pNFP): highlights white matter axons - normal staining in this case, (G) CD34 (H) Ki67: Proliferation index - low in this case (~1%) given its benign nature.

The patient underwent transcortical approach via the middle temporal gyrus for resection of the right hippocampal mass. Pathologic evaluation showed round, uniform cells in an extensively myxoid background with diffuse reactivity for glial fibrillary acidic protein (GFAP), suggesting DNET, World Health Organization (WHO) grade I (Figure 2) as the diagnosis. A post-resection brain MRI without contrast revealed expected post-surgical changes (not shown).

## Discussion

DNETs are low-grade, mixed neuronal-glial neoplasms that are common in the pediatric age group [1]. They were originally described in 1998 by Damas-Duport as a group of supratentorial cortical benign lesions associated with early-onset epilepsy in children and young adults with a slight male predominance [3]. Familiar cases associated with germline fibroblast growth factor receptor 1 (FGFR1) p.R661P mutations have been reported [1].

DNETs usually manifest clinically as chronic, intractable, drug-resistant epilepsy before the age of 20 years and are considered the second most common type of pediatric epileptogenic tumors [1]. These neoplasms have been described in approximately 20% of surgically resected tumors in patients with medically intractable epilepsy [5]. The most common seizure semiology consists of partial complex seizures, followed by generalized tonic-clonic, and simple partial seizures [5].

The tumors typically range from 10 to 25 mm in size and are usually located in the mesial temporal lobe, but frontal and parieto-occipital lobe lesions are also common [5]. The morphology of DNETs varies from well-defined to solitary nodular or poorly demarcated mass lesions [1]. Both computerized tomography (CT) and MRI scans usually show cortical cystic or multicystic lesions. Cystic changes and calcifications are common. Histology is characterized by the presence of small round cells referred to as oligodendroglia-like cells (OLCs) in an abundant mucinous matrix with “floating neurons” without dysplasia [1].

The absence of pathognomonic findings makes the DNET a challenging diagnosis. The differential diagnosis includes other glioneuronal or glial tumors such as gangliogliomas, oligodendrogliomas, pilocytic astrocytomas, and pleomorphic xanthoastrocytomas [5]. However, the majority of OLCs are strongly positive for S-100 protein and Oligo-2, while the “floating neurons” express neuronal markers including synaptophysin neurofilament, NeuN, neuron-specific enolase, MAP2, and class-III beta-tubulin [5]. Given its benign nature is usual to find a low proliferative index Ki67 [6]. This tumor showed diffuse reactivity for GFAP and widely perikaryal synaptophysin reaction, whereas no expression of chromogranin and CD34 was detected. In addition, Ki67 was 1% and expression of phosphorylated neurofilament (pNFP) was normal (Figure 2). All these data led to the diagnosis for a nonspecific DNET.

## Conclusions

DNETs are considered benign, non-recurring mass lesions. They are one of the most common epileptogenic tumors in children and young adults. Usually, patients with DNETs present with chronic drug-resistant and partial complex seizures. One of the challenges for diagnosing DNETs is the absence of pathognomonic findings. This case report showed an atypical presentation due to the presence of asymmetric reflexes, which may be indicative of a possible mass lesion. Complete surgical resection is associated with a seizure-free outcome in 80% to 100% of cases, thus remaining the gold standard therapy.
